# Efficient learning of mixed-state tomography for photonic quantum walk

**DOI:** 10.1126/sciadv.adl4871

**Published:** 2024-03-15

**Authors:** Qin-Qin Wang, Shaojun Dong, Xiao-Wei Li, Xiao-Ye Xu, Chao Wang, Shuai Han, Man-Hong Yung, Yong-Jian Han, Chuan-Feng Li, Guang-Can Guo

**Affiliations:** ^1^ CAS Key Laboratory of Quantum Information, University of Science and Technology of China, Hefei 230026, China.; ^2^ CAS Center for Excellence in Quantum Information and Quantum Physics, University of Science and Technology of China, Hefei 230026, China.; ^3^ Institute of Artificial Intelligence, Hefei Comprehensive National Science Center, Hefei 230031, China.; ^4^ Department of Physics, Southern University of Science and Technology, Shenzhen 518055, China.; ^5^ Hefei National Laboratory, University of Science and Technology of China, Hefei 230088, China.; ^6^ Yangtze Delta Region Industrial Innovation Center of Quantum and Information Technology, Suzhou 215100, China.; ^7^ Institute for Quantum Science and Engineering, Southern University of Science and Technology, Shenzhen 518055, China.

## Abstract

Noise-enhanced applications in open quantum walk (QW) has recently seen a surge due to their ability to improve performance. However, verifying the success of open QW is challenging, as mixed-state tomography is a resource-intensive process, and implementing all required measurements is almost impossible due to various physical constraints. To address this challenge, we present a neural-network–based method for reconstructing mixed states with a high fidelity (∼97.5%) while costing only 50% of the number of measurements typically required for open discrete-time QW in one dimension. Our method uses a neural density operator that models the system and environment, followed by a generalized natural gradient descent procedure that significantly speeds up the training process. Moreover, we introduce a compact interferometric measurement device, improving the scalability of our photonic QW setup that enables experimental learning of mixed states. Our results demonstrate that highly expressive neural networks can serve as powerful alternatives to traditional state tomography.

## Teaser

An efficient mixed-state learning method on the quantum-walk system is demonstrated with an accelerated training process.

## INTRODUCTION

Quantum walk (QW) provides a basic framework for developing effective quantum algorithms and simulating complex phenomena (*1*–*4*). Through interaction with a certain amount of noise, the open (i.e., noisy) QW can produce a remarkable improvement in quantum transport over the noise-free QW (*5*, *6*). Also, such a noise-enhanced feature aids in problem-solving efficiency in tasks such as graph isomorphism testing (*7*), a maze escape (*8*, *9*), and ranking elements (*10*) in large networks. While as a prototypical dynamical process, the open QW can model the dissipative evolution of quantum neural networks (*11*, *12*), and this simulation enables better performance to process various issues such as pattern recognition (*13*). Adding controlled noise into the quantum evolution, QW can be dynamically initialized in any high-dimensional form (*14*–*17*) and generate the Haar random unitary operators (*18*, *19*) required for quantum computation. Implementing the open QW and validating these noise-assisted computational and simulated performances demand a complete density-matrix characterization. However, identifying such a system with inherent high-dimensional structures is indeed a fundamental task to be solved in quantum information science (*20*).

For a discrete-time QW (*21*), the usual local tomographic technique was experimentally demonstrated that enables one to access the two correlated subsystems of coin (*22*–*25*) and position (*26*, *27*), respectively. The tomography was subsequently extended to the walker’s complete wave function with a pure-state hypothesis (*28*) or a low-rank state when decoherence should be estimated (*29*). An alternative tomographic method was recently developed with machine-learning techniques (*30*–*32*), where a trained neural network can be used to recognize and classify QW states (*33*, *34*). The complicated mapping from the measured data to the probability amplitudes can also be learned supervisedly (*35*), and a “physical” wave function of QW is obtained by projecting the unphysical neural-network outputs (*36*). Nevertheless, these methods mainly focus on the tomography of a closed QW, and none of them could be applicable for the mixed density-matrix reconstruction of an open QW, whose number of parameters scales quadratically with the pure-state one.

Here, we experimentally realize the full density-matrix tomography of a one-dimensional (1D) discrete-time QW with arbitrary mixing. We achieve this by parameterizing the complex-valued matrix elements using a neural density operator (NDO) (*37*) because such an effective ansatz has been shown to have better performance than standard maximum likelihood (MaxLik) estimation in terms of the reconstruction fidelity and the number of measurements required. For training NDO to maximize the measurement data likelihood more efficiently, the generalized natural gradient descent (GNGD) procedure, recently developed in our group (*38*), is adopted to achieve about an order of magnitude of convergence rate than traditional gradient descent (GD) procedure. The effective mixed-state tomography of open QW, with a lower requirement of the number of measurements and training iterations for high-fidelity reconstructions, is benchmarked on synthetic datasets. Moreover, we further experimentally demonstrate that the trained NDO can learn noisy quantum states from partial measurements on a photonic QW. Also, the amount of mixing of the QW caused by the interaction with the environment can be well captured.

## RESULTS

### Neural-network ansatz

**Fig. 1.**
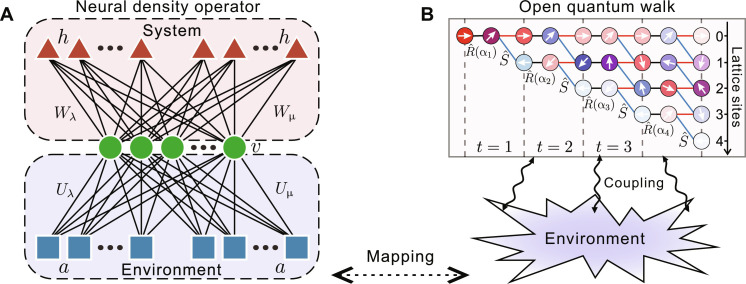
A schematic diagram of NDO and open QW. (**A**) ∣υ〉 is encoded in the visible layer (green circles), a hidden layer *h* (red triangles) captures the correlation within the physical system, and an ancillary layer *a* (blue squares) encodes the coupling between the system and the environment. Matrices *W*_λ,μ_ and *U*_λ,μ_ are the connecting weights. (**B**) Open QW consists of two degrees of freedom, the coin (white arrows) and lattice (colored circles), and the coupling environment introduces the mixing of this system.

The quantum state of open systems is generally described by the density matrix ρ = ∑_υ,υ′_ρ(υ,υ′)∣υ〉〈υ′∣ in a basis ∣υ〉. To obtain the complex-valued matrix elements, a tomography with a series of measurements on a collection of bases {∣υ*^n^*〉} is necessary. The substantial demand for the number of complete tomographic measurements and a complicated data post-processing process make it impractical for a complex system (*20*), whereas neural-network–assisted tomography enables high-fidelity reconstruction of quantum states with fewer measurement resources (*39*). The core step is an effective variational parametrization of the measurement distributions on a quantum state in terms of a neural-network model to infer the complex-valued probability amplitudes (*40*) and the density matrix elements that rely on a linear reconstruction from the trained model distributions (*41*). The linear reconstruction of ρ lacks the positivity constraint such that it cannot give a physical quantum state. To give a physical output, an NDO ansatz can be constructed by purifying the mixed state using an auxiliary Hilbert space *H_a_* to ensure the composite quantum state in the enlarged space ∣Ψ〉 = ∑_υ*a*_Ψ(υ, *a*)∣υ〉∣*a*〉 is pure (*37*). Then, the mixed state of open systems can be written as a reduced state of the composite wavefunction, that is, ρ = Tr*_a_*[∣Ψ〉〈Ψ∣]. The traditional restricted Boltzmann machine as an ansatz of the wave function of the physical system is a two-layer neural network (*39*), including a visible layer υ connected to a hidden one *h* (shown by the top red region in Fig. 1A). Here, to express the composite wave function ∣Ψ〉 in an enlarged space, an additional ancillary layer *a* is imposed as the auxiliary Hilbert space *H_a_* for purifying the mixed state ρ, which corresponds to a fictitious environment mixed with the open systems (herein open QW), as shown by the bottom purple region in Fig. 1A. Then, the probability amplitude of the composite wave function Ψ(υ, *a*) can be parameterized by the three-layer network

$$\Psi_{\lambda \mu }\left(\upsilon ,a\right)=Z_{\lambda }^{-\frac{1}{2}}\sqrt{p_{\lambda }\left(\upsilon ,a\right)}e^{i\text{log}p_{\mu }\left(\upsilon ,a\right)/2}$$ (1)

where $p_{\theta }\left(\upsilon ,a\right)=e^{\sum_{i}\text{log}\left(1+e^{W_{\theta }^{\left[i\right]}\;\upsilon +c_{\theta }^{\left[i\right]}}\right)+a^{T}U_{\theta }\upsilon +b_{\theta }^{T}\upsilon +d_{\theta }^{T}a}$ (θ = {λ, μ}) is the joint distribution of the visible and ancillary layers, mimicking the amplitude and phase of the wave function. *A*^[*i*]^ denotes *i*th row of A, and A^T^ is the transpose of A. Vectors *b*_θ_, *c*_θ_, and *d*_θ_ are the biases coupled to the visible, hidden, and ancillary neurons, respectively. *Z*_λ_ is a normalization constant. The NDO ansatz of the density matrix of the open system can be obtained by tracing out the auxiliary system (*42*–*44*)

$$\rho_{\lambda \mu }=\sum_{\upsilon ,\upsilon^{\prime }}\left[\sum_{a}\Psi_{\lambda \mu }\left(\upsilon ,a\right)\Psi_{\lambda \mu }^{*}\left(\upsilon ,a\right)]\right.|\upsilon \rangle \langle \upsilon^{\prime }|$$ (2)

Thus, the state tomography is mapped into an unsupervised learning task for training the network parameters such that the trained NDO gives an approximate physical state, i.e., ρ_λμ_ ≃ ρ. The expressive ability of the NDO ansatz depends on the number of the hidden and ancillary neurons. We now show how to accomplish the NDO tomography for an open QW.

### Open QW model

Here, we consider a 1D discrete-time QW that is composed of two interacting subsystems of the coin and the lattice, in which the probability amplitude of a quantum walker spreads between lattice sites on a line depending on the internal coin states (*21*). The open version of QW taking into account the effects of the ambient environment is shown in Fig. 1B. The quantum state of open QW is described by a density matrix acting on the product Hilbert space *H* ≡ *H*_C_ ⊗ *H*_*l*_, where $H_{C}\equiv \text{span}\left\{|s\rangle :s=↑,↓\right\}$ and H*_l_*≡ span{∣*l*〉: *l* ∈ ℤ} represent the coin and the lattice subspace, respectively. Then, the reference base is a tensor product of the basis for each subsystem ∣υ⟩=∣s⟩∣l⟩ . Also, the density matrix on this base takes the ⟩ form

$$\rho =\sum_{s,l;s^{\prime },l^{\prime }}\rho_{sl,s^{\prime }l^{\prime }}|s\rangle |l\rangle \langle s^{\prime }|\langle l^{\prime }|.$$ (3)

The dynamics of open QW at each discrete step *t* can be given by (*45*) $\rho_{t+1}=\sum_{k}{\hat{Ε}}_{k}\rho_{t}{\hat{Ε}}_{k}^{†}$ in terms of the Kraus operators ${\hat{Ε}}_{0}=\sqrt{1-\left(w_{s}+w_{l}\right)}\hat{U}$ , ${\hat{Ε}}_{1}=\sqrt{w_{s}}{\hat{\mathbb{P}}}_{s}\hat{U}$ and ${\hat{Ε}}_{2}=\sqrt{w_{l}}{\hat{\mathbb{P}}}_{l}\hat{U}$ . ${\hat{Ε}}_{0}$ contributes to the coherent evolution part and the unitary operator $\hat{U}=\hat{S}\hat{R}$ , where conditional shift operator $\hat{S}=\sum_{l}\left(|↑\rangle \langle ↑|⊗|l+1\rangle \langle l|+|↓\rangle \langle ↓|⊗|l\rangle \langle l|\right)$ and coin-flip operator $\hat{R}\left(\alpha \right)=e^{-i\alpha {\hat{\sigma }}_{y}}{\hat{\sigma }}_{z}$ . ${\hat{\sigma }}_{y}$ and ${\hat{\sigma }}_{z}$ are the Pauli matrices. The ensemble of ${\hat{Ε}}_{1}$ and ${\hat{Ε}}_{2}$ plays a role in eliminating coherence within the physical system, presented in the off-diagonal terms of the density matrix ρ. Projectors ${\hat{\mathbb{P}}}_{s}=\sum_{l}|s,l\rangle \langle s,l|$ and ${\hat{\mathbb{P}}}_{l}=\sum_{s}|s,l\rangle \langle s,l|$ project the walker into the coin state ∣s⟩ and lattice state ∣*l*〉, respectively. The mixing parameter $w=w_{s}+w_{l}\in \left[0,1\right]$ quantifies the transition from the closed QW to the classical random walk (CRW). After an *N*-step walk, the size of the high-dimensional lattice state ∣*l*〉 is *N* + 1. Considering that the coin subspace is modeled as a two-level system, the total dimension of ρ is thus 4(*N* + 1)^2^. Here, we use the NDO ansatz ρ_λμ_ in Eq. 2 as the variational representation of the target QW state ρ in Eq. 3, which is to be reconstructed. Specifically, the visible layer encodes the base ∣υ〉 of dimension 2(*N +* 1) for an *N*-step open QW. The size of the ancillary layer determines the dimension of the fictitious environment Hilbert space used to purify the target QW state. The larger number of hidden and ancillary neurons is needed, enabling the NDO ansatz to capture the increasingly complex correlations within the open QW and between the system and its environment, respectively, as the number of time steps *N* and the mixing parameter *w* increases. Given a series of measurement datasets {*P*(υ*^n^*)} on multiple bases {∣υ*^n^*〉} imposed by the target QW state, the network parameters {*W*_λμ_, *U*_λμ_*, b*_λμ_,* c*_λμ_, *d*_λμ_} are trained on the datasets, such that the reconstructed model distributions approximate the target measurement distributions, i.e., $\left\{P_{\lambda \mu }\left(\upsilon^{n}\right)\right\}=\left\{\rho_{\lambda \mu }^{n}\left(\upsilon^{n},\upsilon^{n}\right)\right\}≃\left\{P\left(\upsilon^{n}\right)\right\}$ . The density matrix $\rho_{\lambda \mu }^{n}=\hat{U}\left(\upsilon^{n},\upsilon \right)\rho_{\lambda \mu }{\hat{U}}^{†}\left(\upsilon^{n},\upsilon \right)$ on the base ∣υ*^n^*〉 is constructed through the base transformation. With the optimized network parameters substituted into Eq. 2, the target mixed state of open QW can be reconstructed by the NDO, i.e., ρ_λμ_ ≃ ρ.

### Experimental implementation

**Fig. 2.**
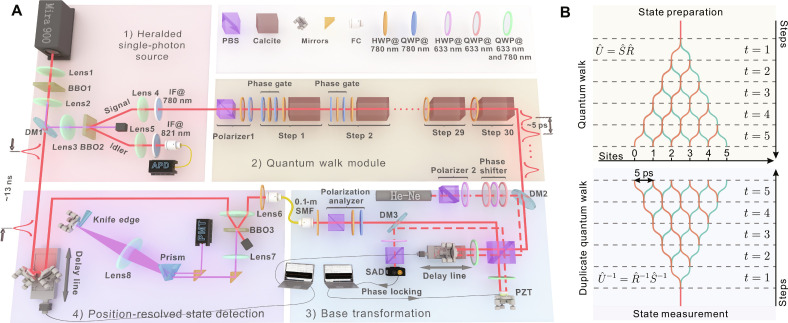
Photonic open QW. (**A**) Experimental setup mainly has four central parts: (1) Spontaneous parameter down-conversion generates the time-correlated photon pairs, where signal photons as the walker and the idler photons serve to herald; (2) the open QW, as reported in the upper panel of (**B**), involves the cascade of HWPs [for operator $\hat{R}\left(\alpha \right)$ ] and calcites (for operator $\hat{S}$ ) that realizes the unitary dynamics ${\hat{U}}^{t}$ . An additional varying phase gate $\hat{R}\left(\beta \right)$ with the QWP-HWP-QWP setting is added to each time step to introduce incoherent contributions; (3) a Michelson interferometer together with a polarization analyzer carries out the base transformation $\hat{U}\left(\upsilon^{n},\upsilon \right)$ on the reference base ∣υ〉, which mimics the effect of a duplicate QW in the lower panel of (**B**). The piezoelectric transducer (PZT)–driven mirror is applied for phase locking by using a reference He-Ne laser, and another moveable mirror is used for adjustable arm length difference; (4) a single-photon frequency upconversion implements the position-resolved detection of the walker in each base. (B) Schematic diagrams show the QW dynamics of a localized initial state (top) and the duplicate QW with time inversion constructed for the state measurements in different bases (bottom), respectively. A list of abbreviations: BBO, β − BaB_2_O_4_; DM, dichroic mirror; IF, interference filter; PBS, polarization-dependent beam splitter; HWP, half-wave plate; QWP, quarter-wave plate; PZT, piezoelectric ceramic; FC, fiber collimator; SMF, single-mode fiber; SAD, Si-amplified detector; PMT, photomultiplier tube; APD, avalanche photodiode detector.

QW can be realized on multiple physical platforms (*46*), and our experimental setup for photonic QW is shown in Fig. 2. More details are given in Materials and Methods. In the experiment, we adopted the heralded single photons as the walker. The coin {∣ ↑ 〉, ∣ ↓ 〉} and the lattice {∣ *l*〉} are encoded in the two polarizations {∣ *H*〉, ∣ *V*〉} and the different arrival times of photons { ∣ *t_l_*〉}, respectively. Polarization flip $\hat{R}\left(\alpha \right)$ is realized by tuning the angle of half-wave plates. A calcite crystal delays ∣*V*〉 by one time-bin length (Δ*t* ~ 5 ps) relative to ∣*H*〉, which realizes the shift operator $\hat{S}$ on the time-bin modes. Considering each state ∣*t_l_*〉 in the time basis is represented by a photonic wave packet with a width of ~140 fs that is far less than the time interval Δ*t*, thus the overlap between different time bins is negligible. Through cascading *N* times, the two operators of $\hat{R}$ and $\hat{S}$ , one can implement an *N*-step walk dynamics. The outgoing *N* + 1 time bins are then injected into a Michelson interferometer for measurement base transformation, followed by an upconverted single-photon detector.

The Michelson interferometer in Fig. 2A, 3 is a core ingredient for full state tomography and eases the usual demand of constructing a duplicate QW system (see Fig. 2B, bottom) to obtain the phase relation between lattice sites (*22*). A moveable mirror in the interferometer introduces a controllable time delay between the two arms, which makes the photons with horizontal polarization ∣*H*〉 travel the integer multiples of 5 ps faster (or later) than those with vertical polarization ∣*V*〉. After the interferometer, a polarization analyzer with a quarter-wave plate (QWP)–half-wave plate (HWP)–polarization-dependent beam splitter (PBS) setting performs the single-qubit Pauli measurements on ${\hat{\sigma }}_{x}$ , ${\hat{\sigma }}_{y}$ , and ${\hat{\sigma }}_{z}$ basis. Therefore, the interferometric measurements between all time bins here can be viewed as the system’s projections on a series of basis { ∣ ↑ 〉 ∣ *l*〉}, { ∣ ↓ 〉 ∣ *l*〉}, $\left\{\frac{1}{\sqrt{2}}\left(|↑\rangle |l\rangle \pm i|↓\rangle |l^{\prime }\rangle \right)\right\}$ , and $\left\{\frac{1}{\sqrt{2}}\left(|↑\rangle |l\rangle \pm |↓\rangle |l^{\prime }\rangle \right)\right\}$ (*l*, *l*′ = 0,1, ⋯, *N*). Moreover, even with the number of steps *N* increasing, the required amounts of the optical elements for these interferometric measurements remain constant thanks to the compact structure. Consequently, the compact interferometer can save 50% of optical resources as there is no need to build another duplicate QW to implement these measurements.

One can classify these projection measurements into a set of bases {∣υ*^n^*〉}. The first base ∣υ^0^〉 is the reference base $|\upsilon \rangle ={\left\{|↑\rangle |l\rangle ,|↓\rangle |l\rangle \right\}}_{l=0}^{N}$, while the bases ∣υ^2*k* − 1^〉 and ∣υ^2*k*^〉 (*k* ∈ {1,2,⋯,*N*+1}) are written as ${\left\{\frac{1}{\sqrt{2}}(|↑\rangle |l\rangle \pm i|↓\rangle |(k-1)]\text{mod}(N+1)\rangle )\right\}}_{l=0}^{N}$ and ${\left\{\frac{1}{\sqrt{2}}\left(|↑\rangle |l\rangle \pm |↓\rangle |\left[l-\left(k-1\right)\right]\text{mod}\left(N+1\right)\rangle \right)\right\}}_{l=0}^{N}$ , respectively. Note that the dividend in the mod function could be negative integers when *l* < *k* − 1, so that the mod function can give values between 0 and *N* but arrange them in different orders for different *k*. Base transformation matrix $\hat{U}\left(\upsilon^{n},\upsilon \right)$ consists of a conditioned shift operator with the periodic boundary (*N* + 1 cycle) ${\hat{S}}^{\prime }=\sum_{l=0}^{N}|↑\rangle \langle ↑|⊗|l\rangle \langle l|+|↓\rangle \langle ↓|⊗|\left(l-1\right)\text{mod}\left(N+1\right)\rangle \langle l|$ and two elementary gates rotating the reference base ${\hat{\sigma }}_{z}$ in coin space to the local base ${\hat{\sigma }}_{x}$ and ${\hat{\sigma }}_{y}$ , respectively. Then, the bases ∣υ^2*k* − 1^〉 and ∣υ^2*k*^〉 can be obtained using the conditioned shift operator ${\left({\hat{S}}^{\prime }\right)}^{k-1}$ together with two elementary gates to act on the reference base ∣υ〉. We note that for an *N*-step QW, *N* times the conditioned shift operator $\hat{S}\prime$ is needed to achieve interferometric measurements between all lattice sites of size *N* + 1.

Throughout the experiment, the probability distribution *P*(υ*^n^*) in each base ∣υ*^n^*〉 is analyzed by using the upconverted detector in Fig. 2A, 4. With a spatial delay line, one can scan a strong pump light of 300 mW to upconvert the signal photons in all time bins with an interval of 5 ps. The upconverted photons are filtered by a spectrum filter that consists of a dispersion prism in the 4-f system to reduce extra scattering noise. Also, the filtered photon counts are then measured by a photomultiplier tube, with which we can obtain experimental distributions *P*_exp_(υ*^n^*). Note that, using the linear reconstruction directly (*41*, *47*), one can only access 2(*N* + 1)^2^ + 2(*N* + 1) matrix elements according to the measurements on these $N_{b}=2\left(N+1\right)+1$ sets of bases. Here, we resort to the effective NDO method to learn the full 4(*N* + 1)^2^ matrix elements from the incomplete measurements, which is an extension of learning some particular properties of quantum states using partial measurements (*48*, *49*).

### Performance of the efficient tomography for QW

Before training the neural network with noisy experimental results, we start by benchmarking the NDO tomography of QW using synthetic datasets (*50*, *51*). For each base ∣υ*^n^*〉, we define a family of 2(*N* + 1) projectors ${\left\{{\hat{\mathbb{P}}}_{j}^{n}=|\upsilon_{j}^{n}\rangle \langle \upsilon_{j}^{n}|\right\}}_{j}$ that satisfy the normalization condition $\sum_{j}{\hat{\mathbb{P}}}_{j}^{n}=I$ (**Ι** is the identity matrix). The projection distribution imposed on the target QW state ρ can be obtained by the linear expression $P\left(\upsilon^{n}\right)={\left\{\text{Tr}\left[{\hat{\mathbb{P}}}_{j}^{n}\rho ]\right]\right\}}_{j}$ , with ∑_υ*^n^*_*P*(υ*^n^*) = 1. For an *N*-step QW, we generate the synthetic datasets {*P*(υ*^n^*)} in a collection of $N_{b}=2\left(N+1\right)+1$ bases {∣υ*^n^*〉} to train the neural network. The training process minimizes the cost function, defined as the total statistical distance $D_{\theta }=\sum_{n}\sum_{\upsilon^{n}}P\left(\upsilon^{n}\right)/\text{log}\left[P\left(\upsilon^{n}\right)/P_{\theta }\left(\upsilon^{n}\right)\right]$ , between the target and the reconstructed distributions. The process starts with the network parameters being initialized to random values, and in each optimization iteration *i*, the parameters are updated according to the GNGD procedure (*38*): $\theta_{i+1}=\theta_{i}-\eta_{i}G^{-1}\nabla D_{\theta }$ , where the searching step η*_i_* is determined by the line search process. To determine a well-performed metric *G* that can speed up the gradient-based optimization, the core idea of GNGD is to introduce a proper reference space, which is regarded as a flat space here. By choosing the identity matrix as the metric of reference space $G_{\mathrm{ij}}^{\text{ref}}=\delta_{i,j}$ , the metric for the cost function *G* can be obtained through the conversion of coordinates (See Materials and Methods for details). All the NDO reconstructions given below were obtained in this way.

**Fig. 3.**
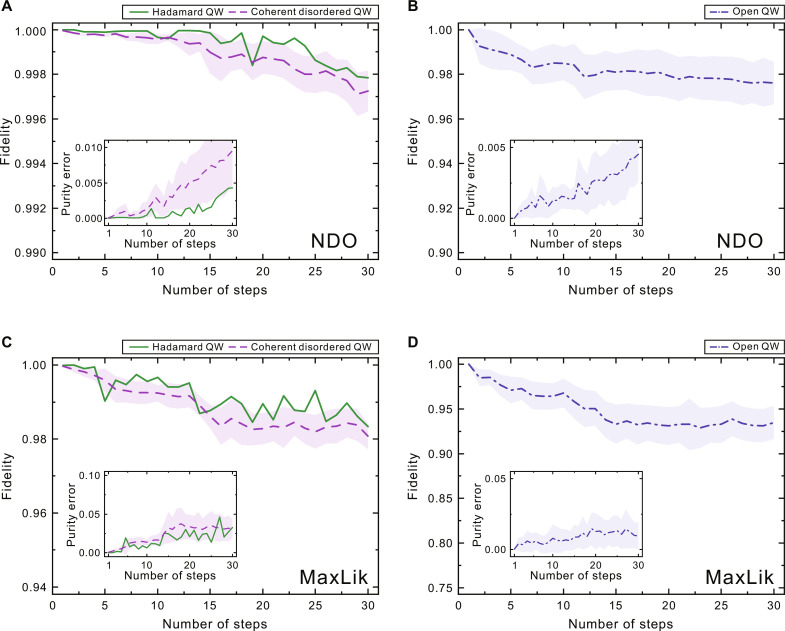
Benchmarking NDO and MaxLik tomography using partial measurements. (**A** and **B**) NDO reconstruction fidelity as a function of the number of time steps for Hadamard QW with α ≡ π/4 (green solid line), coherent disordered QW with α*_t_* being time dependent (red dashed line), and open QW with arbitrary mixing (blue dashed dotted line). The insets show the error in the purity of the reconstructed states ρ_λμ_ for each time step. The shaded regions for the three types of QW are the SEs of NDO reconstruction with 100 random samples for each step, and the lines are the averaging results. (**C** and **D**) Corresponding results for the MaxLik method.

All the initial state is set to be ρ_0_ = ∣ψ_0_〉〈ψ_0_∣ with $|\psi_{0}\rangle =\frac{1}{\sqrt{2}}(|↑\rangle +i|↓\rangle )⊗|0\rangle$ . We first focus on the simplest model where the flip angle *α* is time independent and the mixing parameter *w* = 0. Consider that the coin operator is a Hadamard gate (α = π/4), and the corresponding walk is the typical Hadamard QW. The fidelity of the NDO tomography performed on the synthetic datasets is reported by the green solid line in Fig. 3A, and a uniformly high fidelity is achieved even with an increasing number of time steps. In a bit complicated case, the angle α ∈ [0, π) is set to be time dependent and becomes completely random for each step. For a given step of this coherent disordered QW (*52*, *53*), 100 disordered configurations are generated, corresponding to 100 different QW dynamics of the quantum states. The red dashed line in Fig. 3A shows the mean performance of the reconstruction fidelity obtained by averaging over the results of the 100 samples for each time step. A reconstruction fidelity error below 4 × 10^−3^ was found for the coherent disordered walk with 3000 samples in total.

We then turn to a more challenging scenario of open QW interacting with a simulated environment (*19*, *54*). Decoherence can be introduced by inserting an extra phase gate $\hat{R}\left(\beta \right)=e^{\frac{i}{2}\beta {\hat{\sigma }}_{z}}$ with a fast fluctuating phase β ∈ [ − δβ, δβ] into each step of coherent QW. The fluctuating degree δβ ∈ [0, π] can effectively add different degrees of decoherence such as the mixing parameter *w* ∈ [0,1] in the open quantum-walk model because the results obtained for a certain value of the mixing parameter w can be faithfully reproduced with a proper fluctuating degree δβ (*50*). Specially, δβ = 0 corresponds to a coherent QW with *w* = 0, and δβ = π results in a fully decoherent CRW with *w* = 1. We also generated 100 samples of the open QW with different mixing for each time step, and the NDO reconstruction fidelity $F\left(\rho ,\rho_{\lambda \mu }\right)=\mathrm{Tr}{\left(\sqrt{\sqrt{\rho }\rho_{\lambda \mu }\sqrt{\rho }}\right)}^{2}$ shown in Fig. 3B can still reach a high value of ∼97.5% with the partial measurements. While this plot and our experiment below are for the example of the phase decoherence of the coin, we note that the NDO tomography is also valid for the open QW with depolarizing noise (see the “Benchmarking neural-network reconstruction of an open QW with depolarizing noise” section in the Supplementary Materials) (*50*). Beyond fidelity, an important question is whether the mixing of QW is reproduced accurately in the NDO reconstruction. The insets in Fig. 3 (A and B) display the reconstructed error in the purity $|\mathrm{Tr}\left[\rho_{\lambda \mu }^{2}\right]-\mathrm{Tr}\left[\rho^{2}\right]|$ for the three scenarios. The mean value of the purity error is lower than 5 × 10^−3^, 1 × 10^−2^, and 5 × 10^−3^ for the Hadamard, coherent disordered, and open QWs, respectively, finding a close agreement with the expected results of the target state ρ.

All the training above for the open QW is implemented in a FORTRAN program, and the corresponding code can be found in (*51*). For the neural-network hyperparameters, the number of visible neurons is uniquely determined by the dimension of the basis ∣υ^0^〉 = ∣*s*〉∣*l*〉. The number of neurons in the hidden and visible layers is fixed to be 10 for each time step of the Hadamard QW and coherent disordered QW in Fig. 3A and 15 for the model of open QW in Fig. 3B, respectively. In each iteration process, we directly sum over all the terms in $\nabla D_{\theta }$ for being free of the sampling error of Monte Carlo sampling. The criterion used to stop training is that the norm of the gradient $\nabla D_{\theta }$ reaches a fixed value of 10^−8^, and in general, the training stopped after 100 to 2000 iterations, depending on the dimension of the target density matrix and the choice of model hyperparameters of the neural network.

As a comparison, we performed standard MaxLik estimation for the three types of QW. The MaxLik estimator we use takes the form ρ*_m_* = *TT*^†^/Tr(*TT*^†^). The matrix *T* is a lower triangular matrix with diagonal elements being real-valued and nondiagonal elements being complex-valued. For an *N*-step open QW, 4(*N* + 1)^2^ real numbers need to be optimized, such that the MaxLik can give a distribution $P_{m}\left(\upsilon^{n}\right)={\left\{\mathrm{Tr}\left[{\hat{\mathbb{P}}}_{j}^{n}\rho_{m}\right]\right\}}_{j}$ approximately equal to the measurement one $P\left(\upsilon^{n}\right)={\left\{\mathrm{Tr}\left[{\hat{\mathbb{P}}}_{j}^{n}\rho \right]\right\}}_{j}$ (*n* = 0,1, ⋯,2(*N* + 1)) in terms of the total statistical distance as the cost function. The values of the 4(*N* + 1)^2^ real numbers are randomly initialized and updated using the conjugate gradient (CG) algorithm (*55*). The stopping criterion is that the norm of the gradient reaches a fixed value of 10^−8^. The reconstruction fidelity and purity error for three types of QWs are shown in Fig. 3 (C and D). We find that, for the three scenarios, the performance of the NDO learning method is superior to that of the standard MaxLik method when the given measurement is partial, which is consistent with previous works (*37*, *56*–*58*).

### Experimental mixed-state reconstructions

**Fig. 4.**
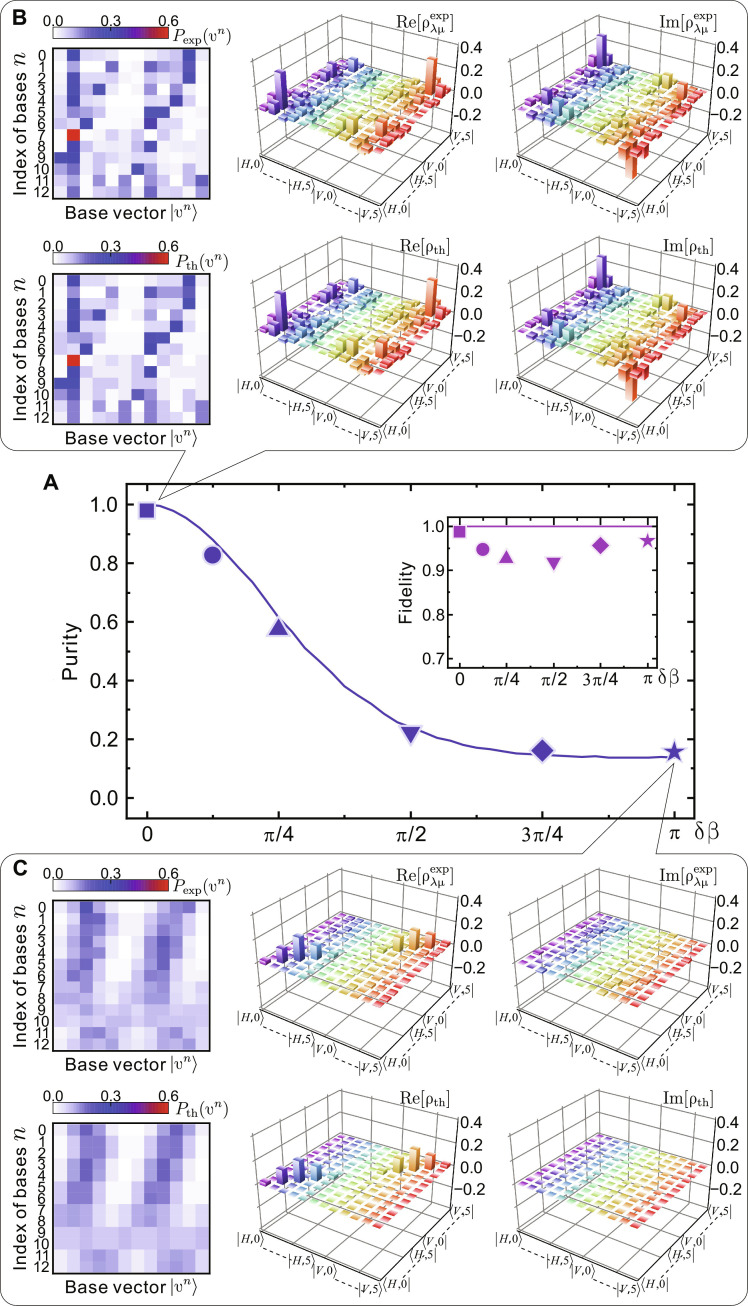
Experimental NDO tomography of open QW in a simulated environment. (**A**) Purity of the NDO reconstructed states $\rho_{\lambda \mu }^{\text{exp}}$ (blue symbols), trained on the experimental measurement data *P*_exp_(υ*^n^*) for a five-step QW with six distinct mixings. The red symbols in the inset report the reconstruction fidelity. The blue and red solid lines give the theoretical results of target states ρ_th_. Error bars considering the statistical noise are smaller than the symbol size. (**B** and **C**) Measured probability distributions *P*_exp_(υ*^n^*) on 13 sets of bases, real and imaginary parts of $\rho_{\lambda \mu }^{\text{exp}}$ (from left to right) for the Hadamard QW with δβ = 0, and the fully decoherent walk with δβ = π, respectively. The bottom panels in (B) and (C) represent the corresponding theoretical expectations.

After benchmarking it on synthetic data, we now demonstrate the efficient learning of open QW with noisy experimental results. Using Polarizer1 in Fig. 2A, 2, the walker’s initial state is fixed to be a product state $\frac{1}{\sqrt{2}}\left(|H\rangle +i|V\rangle \right)⊗|t_{l=0}\rangle$ . The first experiment we performed was a five-step Hadamard walk interacting with a controllable simulated environment. Besides a Hadamard gate realized by an HWP with its optical axis oriented at 22.5°, the extra phase gate $\hat{R}\left(\beta \right)=|H\rangle \langle H|+e^{i\beta }|V\rangle \langle V|$ is introduced by a configuration of QWP-HWP-QWP for each time step. The configuration features two QWPs rotated to an angle of 45° and a sandwiched HWP whose rotation angle controls the relative phase β between horizontal and vertical polarization (*59*). To mimic the decoherence effect, the five sandwiched HWPs are installed on the motorized stages to constantly change their rotation angles with a controlled fluctuating degree δβ = 0, π/8, π/4, π/2,3π/4 and π. Then, an ensemble measurement of $P_{\text{exp}}\left(\upsilon^{n}\right)$ is performed on $N_{b}=13$ sets of bases by unitizing the Michelson interferometer (see Fig. 2A, 3) and the upconverted detector (see Fig. 2A, 4). As reported in Fig. 4A, NDO can learn to reproduce the purity of open QW’s mixed states present in the experimental data well. These results allow us to investigate the QW-to-CRW transition in greater depth than by only considering their transport behaviors (*60*) exhibited in the diagonal terms of the density matrix. For the Hadamard walk (*w=0*) shown in Fig. 4B, beyond the typical characterization with pronounced side peaks, the underlying coherence is clearly presented in the complex-valued off-diagonal matrix elements of $\rho_{\lambda \mu }^{\text{exp}}$ . The decoherence introduced by the simulated environment can completely destroy the phase relation between lattice sites such that CRW (*w* = 1) dominates. Consequently, it can bring the off-diagonal matrix elements to zero and cause the diagonal elements to have a classical binomial distribution, as shown in Fig. 4C.

As shown in the inset of Fig. 4A, the fidelity of the reconstructed full density matrix is greater than 0.919 and is superior to the one using the MaxLik method (>0.835). The deviation in the reconstruction fidelity between the experimental and synthetic datasets mainly results from systematic errors present in the experiment. The two experimental errors consist of an inexact orientation of the wave plates with a precision of ±0.2° with respect to the correct angle and an inexact calibration of the QW interference network step-by-step with an accumulated phase error of ±0.01π per step. We note that when the fluctuating degree δβ = 0 and π, the target QW state ρ is a rank-one density matrix and a completely mixed state, respectively. NDO only needs to learn the correlation within the physical system (captured by the hidden layer) or the correlation between the system and the ambient environment (captured by the ancillary layer). By contrast, when δβ = π/2, NDO needs to learn both the complex correlations within the physical system and between the system and the ambient environment. This could lead to the reconstruction fidelity at δβ = π/2 being slightly lower than the scenarios with δβ = 0 and π.

**Fig. 5.**
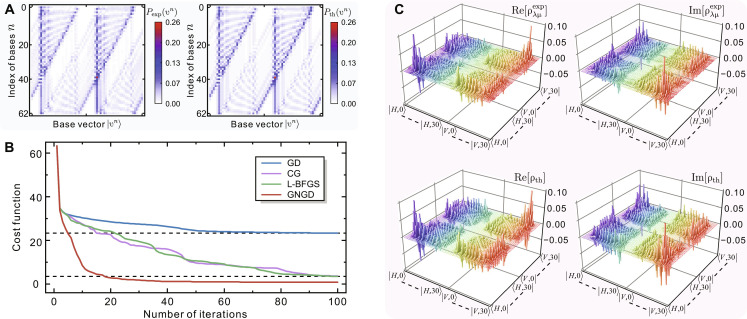
Experimental GNGD-enhanced NDO tomography of QW in a real environment. (**A**) Measured probability distributions *P*_exp_(υ*^n^*) on 63 sets of bases for a 30-step Hadamard walk. The right panel is the theoretical expectation. (**B**) Cost function *D*_θ_ versus the number of training iterations for NDO tomography using GD, CG, L-BFGS, and GNGD optimizer. (**C**) Real and imaginary parts of the NDO reconstructed state $\rho_{\lambda \mu }^{\text{exp}}$ , and the theoretical expectations are shown in the bottom panels.

As the total QW step *N* increases, the learning of mixed states with the dimension of 4(*N* + 1)^2^ demands the complicated training of a larger neural network to keep its high expressive power. Thus, we introduce the GNGD procedure (*38*) to enhance the training efficiency of NDO. To better demonstrate the performance of GNGD for addressing complex networks of NDO, we extend the number of discrete time steps *N* = 30, and a 30-step Hadamard walk is experimentally realized. Interferometric measurements on $N_{b}=63$ sets of bases are performed, and the obtained distributions *P*_exp_(υ^n^) are displayed in Fig. 5A. The mean value of classical indicator similarity of the measured distributions, defined as $S=\sum_{n}\sum_{\upsilon^{n}}\sqrt{P_{\text{exp}}\left(\upsilon^{n}\right)P_{\mathrm{th}}\left(\upsilon^{n}\right)}/N_{b}$ , reads 0.965 ± 0.008, giving good agreement with the theoretical predictions. In Fig. 5B, we compare the cost function $D_{\theta }$ as a function of the number of iterations using GD, CG (*55*), Limited-memory Broyden-Fletcher-Goldfarb-Shanno (L-BFGS) (*61*), and GNGD methods for training NDO, respectively. It can be found that the GNGD optimizer enables a lower value of $D_{\theta }$ in one order of magnitude fewer iterations than the traditional GD-based optimization and achieves a fivefold speedup in the training process over CG and L-BFGS. As shown in Fig. 5C, the GNGD-enhanced NDO can efficiently output the experimental mixed state $\rho_{\lambda \mu }^{\text{exp}}$ with 62^2^ complex-valued matrix elements using fewer training iterations and partial measurements. The emblematic side peaks and the off-diagonal elements that represent coherence are still clear after the 30-step Hadamard walk. The reconstructed fidelity and purity are 0.789 ± 0.011 and 0.675 ± 0.013, respectively. A test on synthetic data yields the values of fidelity and purity both greater than 0.995, indicating that the uncontrolled decoherence introduced by the real environment is the major deviation for the experimental reconstruction. While classical similarity indicates how much two distributions overlap, the fidelity we obtained is a true estimator of the quality of the quantum states produced, characterizing the quality of experimental quantum systems more accurately.

## DISCUSSION

In this work, we have experimentally demonstrated an effective learning of mixed states for open photonic QW in terms of the number of training iterations, the amount of measurement resources, and the reconstruction fidelity. The learning method is achieved by training a neural-network–parameterized density operator on measurement datasets and then outputting complex-valued matrix elements accelerated by the GNGD procedure. Although several works have already demonstrated the power of various neural networks for learning the mixed state of open systems, these proposals are mainly focused on the reconstruction of a single qubit (*62*), a single qudit (*63*), and the dominant eigenstates of a low-rank mixed state (*64*). Especially, the effectiveness of NDO tomography was demonstrated only in a simple two-qubit open system (*37*). Until recently, the tomography of the density matrix of dimensions up to 32^2^ has been reported using the restricted Boltzmann machine (*56*) and the conditional generative adversarial network (*57*, *58*). By contrast, we both theoretically and experimentally realized the NDO tomography of a 2 × *n* open QW system and the reconstruction of the mixed state with the largest number of matrix elements, up to 62^2^, surpassing all previously reported results. Furthermore, this experimental study is also the first time to verify the effectiveness of our self-developed GNGD optimization algorithm (*38*) for accelerating training on the long-standing challenging task of experimental learning of open QW. The GNGD optimizer can be applied to various neural-network architectures for effectively speeding up the gradient-based tomography of the quantum state (*65*, *66*) and the quantum process (*67*–*69*).

In addition to the neural-network ansatz, an effective mixed-state tomography has recently been reported using the tensor-network ansatz (*70*). Compared to the locally purified state ansatz there, the correlations captured by the hidden and ancillary layers in NDO are inherently nonlocal. Therefore, the NDO ansatz we use is better suited to describe the 2 × *n*-dimensional open QW with inherent high-dimensional structures (*39*). To capture the complex correlations within the physical system and the environment more accurately as the time step increases, we usually increase the number of hidden and ancillary neurons, respectively. One can introduce the deep restricted Boltzmann machine with improved representational power (*71*), at the cost of growing training complexity, to further develop the NDO method to learn the open quantum systems. Adding an extra noise layer (*40*) or using global measurements (*72*, *73*) could improve the robustness of state reconstructions against experimental errors. Overall, our approach provides a promising avenue for addressing the challenges associated with verifying open QW. We expect that these results will lead to new insights and discoveries in this exciting area of research on numerous physical systems such as fiber loop (*74*), spatial path (*75*), orbital angular momentum (*76*), transverse momentum (*77*, *78*), hybrid architecture (*79*), etc. Moreover, the full quantum state tomography technique can be combined with the known abilities of arbitrary initialization (*14*, *24*) and flexible manipulation, which can inspire a prospective QW platform for developing diverse applications in a range of fields.

## MATERIALS AND METHODS

### Training the neural network by GNGD

The training of the neural network is to learn the optimal parameters θ = {λ, μ} that minimize the cost function $D_{\theta }$. The derivative of the cost function with respect to the network parameters reads

$$\begin{array}{c} \nabla D_{\theta }=-\sum_{n}\sum_{\upsilon^{n}}\frac{P\left(\upsilon^{n}\right)}{\rho_{\theta }^{n}\left(\upsilon^{n},\upsilon^{n}\right)}\\ \sum_{\alpha ,\beta }U^{n}\left(\upsilon^{n},\alpha \right)\rho_{\theta }\left(\alpha ,\beta \right)\nabla A_{\theta }\left(\alpha ,\beta \right)U^{n,†}\left(\beta ,\upsilon^{n}\right)+\\ N_{b}\sum_{\upsilon }\rho_{\theta }\left(\upsilon ,\upsilon \right)\nabla A_{\theta }\left(\upsilon ,\upsilon \right) \end{array}$$ (4)

where $\rho_{\theta }^{n}\left(\upsilon ,\upsilon^{\prime }\right)=\sum_{\alpha ,\beta }U^{n}\left(\upsilon ,\alpha \right)\rho_{\theta }\left(\alpha ,\beta \right)U^{n,†}\left(\beta ,\upsilon^{\prime }\right)$ , $U^{n}=\langle \upsilon^{n}|\upsilon \rangle$ is the base transformation matrix from the reference base ∣υ〉 to ∣υ*^n^*〉, $N_{b}$ is the total number of measurement bases. $\rho_{\lambda \mu }\left(\alpha ,\beta \right)=Z_{\lambda }^{-1}e^{A_{\lambda \mu }}$ is the rebuild density matrix of the system, where

$$\begin{array}{c} A_{\lambda \mu }\left(\upsilon ,\upsilon \prime \right)=\Gamma_{\lambda }^{+}\left(\upsilon ,\upsilon \prime \right)+i\Gamma_{\mu }^{-}\left(\upsilon ,\upsilon \prime \right)+\Pi_{\lambda \mu }\left(\upsilon ,\upsilon \prime \right),\\ \Gamma_{\theta }^{\pm }\left(\upsilon ,\upsilon \prime \right)=\frac{1}{2}\left[\sum_{i}\text{log}\left(1+e^{W_{\theta }^{\left[i\right]}\upsilon +c_{\theta }^{\left[i\right]}}\right)\pm \sum_{i}\text{log}\left(1+e^{W_{\theta }^{\left[i\right]}\upsilon \prime +c_{\theta }^{\left[i\right]}}\right)+b_{\theta }^{T}\left(\upsilon \pm \upsilon \prime \right)\right],\\ \Pi_{\lambda \mu }\left(\upsilon ,\upsilon \prime \right)=\sum_{i}\text{log}\left\{1+\text{exp}\left[\frac{1}{2}U_{\lambda }^{\left[i\right]}\left(\upsilon +\upsilon \prime \right)+\frac{i}{2}U_{\mu }^{\left[i\right]}\left(\upsilon -\upsilon \prime \right)+d_{\lambda }^{\left[i\right]}\right]\right\} \end{array}$$ (5)

The superscript [*i*] means the *i*th row elements. The network parameters to be optimal here are {*W*_θ_, *U*_θ_, *b*_θ_, *c*_θ_, *d*_θ_}. *W*_θ_ and *U*_θ_ are the weight matrices, whose dimension is *m* × *d* with *d* being the degree of freedom of the physical system and *m* being the number of hidden or ancillary neurons. *b*_θ_, *c*_θ_, and *d*_θ_ are the vectors of dimension *d* representing the biases coupled to the visible, hidden, and ancillary neurons, respectively. To accelerate the convergence efficiency of training, the GNGD method emphasizes introducing a proper reference space (*38*). Here, the space span by the density matrix ρ_θ_(α, β) is selected as the reference manifold. We choose a simple identity matrix $G_{\alpha ,\beta ;\alpha^{\prime },\beta^{\prime }}^{\mathrm{ref}}=\delta_{\alpha ,\alpha^{\prime }}\delta_{\beta ,\beta^{\prime }}$ as the metric of reference space. Through the conversion of coordinates, the metric *G* for the cost function can be determined as

$$G_{i,j}=\sum_{\alpha ,\beta ;\alpha^{\prime },\beta^{\prime }}\frac{\partial \rho_{\theta }\left(\alpha ,\beta \right)}{\partial \theta_{i}}G_{\alpha ,\beta ;\alpha^{\prime },\beta^{\prime }}^{\mathrm{ref}}\frac{\partial \rho_{\theta }\left(\alpha^{\prime },\beta^{\prime }\right)}{\partial \theta_{j}}$$ (6)

With this metric, the network parameters are updated according to the gradient-based optimization as

$$\theta_{i+1}=\theta_{i}-\eta_{i}G^{-1}\nabla D_{\theta }$$ (7)

where η_*i*_, which is determined by the line search process, is the searching step at iteration *i*.

### Heralded single-photon source

A Ti: sapphire laser source (Mira 900, Coherent) launches a series of optical pulses with a temporal pulse width of 140 fs, a repetition rate of 76 MHz, the wavelength λ = 800 nm, and an average power of 1 W. The ultrafast optical pulses focused by Lens1 (focal length *f* = 100 mm) pump the first β − ΒaB_2_*O*_4_ (BBO1), causing the second harmonic generation. The frequency-doubled pulses with horizontal polarization and the residual fundamental pulses with vertical polarization are collimated by Lens2 and then spatially separated by the first dichroic mirror (DM1). The fundamental pulses go through a pair of cylinder lenses to reform the beam’s profile into a Gaussian shape and are then reused for position-resolved state detection. The frequency-doubled pulses with λ = 400 nm and an average power of 200 mW are used as a pump source and focused on the BBO2 by Lens3 (focal length *f* = 100 mm). Here, BBO2 is designed for type-II, nondegenerate, and “beam-like” spontaneous parametric down conversion (SPDC). The generated time-correlated photon pairs (namely, signal photons with λ = 780 nm and horizontal polarization and idler photons with λ = 821 nm and vertical polarization) are spatially separated by the noncollinear SPDC process with a half opening angle of 3° . The idler photons, collimated by the Lens5 and cleaned by a spectrum filter, are coupled to a single-mode fiber (SMF) and then detected by the avalanche photodiode detector (SPCM-AQRH-14-FC, Excelitas) to herald the appearance of the signal photons. The coincidence count rate between idler and signal photons is 4.5 × 10^2^ pairs/(s mW). The collimated and filtered signal photons are adopted as the walker and sent to the QW module.

### Time-multiplexing photonic QW

The time-multiplexing QW is achieved by encoding the walker’s lattice and coin space in the arrival time and polarization of signal photons, respectively. The signal photons at the input of the QW module are set to be in the first time bin, i.e., the origin in lattice space with *l* = 0. Then, the initial polarization state of signal photons $|{↑}_{y}\rangle =\frac{1}{\sqrt{2}}\left(|H\rangle +i|V\rangle \right)$ is prepared through a PBS, a HWP, followed by a QWP. ∣*H*〉 and ∣*V*〉 represent horizontal and vertical polarization, respectively. Thus, the initial state of the walker is ∣ψ_0_〉 = ∣*l* = 0〉 ⊗ ∣↑*_y_*〉. In the protocol of Hadamard QW, the unitary operator of a single step $\hat{U}=\hat{S}\hat{R}$ is composed of an HWP rotated to a specific angle at 22.5° (for operator $\hat{R}$ ) and a calcite crystal (for operator $\hat{S}$ ). Each piece of calcite is cut parallel to its optical axes to a length of 8.98 mm, whose birefringence Δ = 0.167 at λ = 800 nm will induce a 5-ps time shift between the horizontal ∣*H*〉 and vertical polarization ∣*V*〉 of the signal photons. In the experiment, *N* sets of HWPs and calcite crystals are positioned to realize an *N*-step Hadamard walk. Thus, the lattice space of the walker at the end of the QW module consists of the superposition of *N* + 1 time bins with a time interval of 5 ps. Also for each lattice site, the walker has an internal coin state, which is usually distinct at a different site. A QWP-HWP-QWP setting for realizing the phase gate operator $\hat{R}\left(\beta \right)=e^{\frac{i}{2}\beta {\hat{\sigma }}_{z}}$ can be introduced at each QW step to mimic a controlled decoherence effect. Two QWPs are rotated to an angle at 45^°^. HWP in the setting is installed on the motorized stage (PR50PP, Newport) to change its rotation angle constantly. The fluctuating range [ − δβ, δβ] determines the degree of decoherence and has a similar effect as the mixing parameter *w*: δβ = 0 is the coherent QW with *w* = 0, and δβ = π results in a fully decoherent classical walk with *w* = 1.

### Base transformation and phase-locking technique

The base transformation is achieved by a Michelson interferometer consisting of a PBS, two QWPs rotated to an angle at 45°, and two mirrors. One mirror is piezoelectric ceramic (piezoelectric transducer)–driven to compensate for the phase fluctuation of the interferometer, and the other mirror is installed on a motorized positioning system (KXL06100, Suruga). The moveable mirror is controlled to induce a time shift between the two arms of the interferometer, which is accurately the integer multiples of 5 ps for ensuring maximal visibility between different time bins of the signal photons and avoiding temporal mode mismatching. The phase difference between the two arms can be controlled by the phase shifter (PS) and locked in a fixed value by an ancillary Helium-Neon (He-Ne) laser using a proportional-integral-differential (PID) feedback unit (*80*). The PS is the configuration of QWP-HWP-QWP, where two QWPs are rotated to an angle at 45° and the rotation angle of HWP controls the relative phase between ∣*H*〉 and ∣*V*〉 states. Initially, the He-Ne laser source (HNL050LB, Thorlabs) with λ = 632.8 nm is prepared in the state $|{↓}_{x}\rangle =\frac{1}{\sqrt{2}}\left(|H\rangle -|V\rangle \right)$ through the Polarizer2 and then launched to the same propagation path with the signal photons through DM2. The signal photons and the He-Ne laser pass through the interferometer and are spatially separated by DM3. The reflected He-Ne laser is projected in the $|{↑}_{x}\rangle =\frac{1}{\sqrt{2}}\left(|H\rangle -|V\rangle \right)$ state and detected by an amplified Si photodetector (PDA8A/M, Thorlabs). The detected He-Ne laser intensity is initially set near a reference value through PS. Within the acquisition time of time-correlated photon pairs, the PID unit compares the detected intensity using the amplified Si photodetector to the reference value and then uses the difference to calculate a new input value through the PID algorithm that is designed to keep the detected intensity at the reference value. Thus, the unstable phase difference of the interferometer for signal photons can then be locked with the He-Ne laser. PS can be used to compensate the phase difference of the signal photons introduced by the two arms of the interferometer to zero. After passing through the Michelson interferometer and the polarization analyzer, the signal photons are collected into a 0.1-m-long SMF for spatial filtering. The two HWPs at the input and output of the SMF are used for polarization maintenance.

### Frequency upconversion single-photon detector

To measure the probability distribution *P*(υ*^n^*) in each base ∣υ*^n^*〉, a position-resolved detection of the signal photons is constructed. The lattice space of the signal photons consists of a pulse train with a time interval of 5 ps. Here, we use a single-photon frequency upconversion system converting the high time resolution to the spatial resolution. Specifically, the residual fundamental pulses with λ = 800 nm and average power of 300 mW during the second harmonic generation serve as the strong pump pulse. The upconverted photons are generated in BBO3, where the signal photons and the strong pump pulse meet, and then detected by a photomultiplier tube (H10682, Hamamatsu). In the upconversion process, the propagation path of the strong pump pulse is delayed and aligned using two moveable mirrors installed on a motorized positioning system (KXL06100, Suruga). Also, the alignment of the signal photons is achieved by replacing them with a reference coherent laser to optimize the spatial mode matching. In addition, the delay of the strong pump pulse is fine-tuned to match each pulse in the signal-photon pulse train in the time domain. A spectrum filter composed of a dispersion prism is used to reduce the background.
