# Supplementary material for: Efficient learning of mixed-state tomography for photonic quantum walk
Source: Sci Adv. 2024 Mar 15;10(11):eadl4871. doi: 10.1126/sciadv.adl4871 (PMC12697567; doi:10.1126/sciadv.adl4871)
Supplement: Supplementary file 1 — Supplementary Text Figs. S1 and S2 [file sciadv.adl4871_sm.pdf]

Science Advances
AAAS

Supplementary Materials for

Efficient learning of mixed-state tomography for photonic quantum walk

Qin-Qin Wang *et al.*

Corresponding author: Xiao-Ye Xu, xuxiaoye@ustc.edu.cn; Man-Hong Yung, yung@sustech.edu.cn; Yong-Jian Han, smhan@ustc.edu.cn; Chuan-Feng Li, cfli@ustc.edu.cn

Sci. Adv. **10**, eadl4871 (2024)
DOI: 10.1126/sciadv.adl4871

This PDF file includes:

Supplementary Text
Figs. S1 and S2

Supplementary Text

Section A. Neural-network ansatz.

Traditional pure-state ansatz. – For a quantum system in a pure state, the density operator characterizing the system takes the rank-one form $\rho = |\psi\rangle\langle\psi|$, where the wavefunction $|\psi\rangle = \sum_v \psi(v)\,|v\rangle$ is generally described by a set of complex probability amplitudes:

$$\psi(v) = \sqrt{p(v)}e^{i\phi(v)} \tag{S1}$$

in a basis $|v\rangle$. In this case, it has been shown that a restricted Boltzmann machine (RBM) can be an effective ansatz of the system's wavefunction in arbitrary dimensions (39, 40, 61). The standard RBM is a two-layer neural network with binary-valued neurons that interact only between the adjacent layers but not intralayer, where a visible v encodes the basis of the Hilbert space $|v\rangle$ and a hidden layer h with nonlocal connections to the visible one encodes the complex correlations within the physical systems. The network parameters of the RBM are given by $\theta = \{W_\theta, b_\theta, c_\theta\}$, which consists of the connecting weight matrices W_θ and bias vector b_θ (c_θ) coupled to the visible (hidden) layer. In terms of the set of neural parameters, the Ising-type energy is defined by:

$$E_\theta(v,h) = -\sum_{i,j} W^\theta_{ij} h_i v_j - \sum_j b^\theta_j v_j - \sum_i c^\theta_i h_i. \tag{S2}$$

The Boltzmann distribution of the energy reads as:

$$p_\theta(v,h) = \frac{1}{Z_\theta} e^{-E_\theta(v,h)}, \tag{S3}$$

where Z_θ is a normalization constant. To describe the complex amplitude $\psi(v) = \sqrt{p(v)}e^{i\phi(v)}$ in the basis $|v\rangle$, two separate RBMs with parameter sets $\lambda = \{W_\lambda, b_\lambda, c_\lambda\}$ and $\mu = \{W_\mu, b_\mu, c_\mu\}$ are utilized to express the amplitude and phase, respectively, which are expressed as:

$$\psi_{\lambda\mu}(v) = Z_\lambda^{-\frac{1}{2}} \sqrt{p_\lambda(v)} e^{i \log p_\mu(v)/2}, \tag{S4}$$

where $p_\lambda(v)$ and $p_\mu(v)$ are the marginal distributions over visible neurons:

$$p_\lambda(v) = \frac{1}{Z_\lambda} \sum_h e^{-E_\lambda(v,h)}, \qquad p_\mu(v) = \sum_h e^{-E_\mu(v,h)}. \tag{S5}$$

The learning of the RBM involves finding the optimal set of neural parameters $\theta = \{\lambda, \mu\}$ such that the model distribution can perfectly match the experimentally measured distribution in a collection of bases $\{|v^n\rangle\}$, i.e., $P_{\lambda\mu}(v^n) = |\psi_{\lambda\mu}(v^n)|^2 \simeq |\psi(v^n)|^2 = P(v^n)$. By plugging the optimal neural parameters into Eq.(S4), the target wavefunction can be reconstructed.

Mixed-state ansatz. – In realistic scenarios in which the purity loss of the physical system is inevitable, the quantum states of interest are often mixed because the system is barely isolated from its ambient environment. The mixed states of the open quantum systems can be characterized by the density operator:

$$\rho = \sum_{v,v'} \rho(v,v') \left|v\right\rangle \left\langle v'\right| \tag{S6}$$

in a basis $|v\rangle$, which should be Hermitian $\rho^\dagger = \rho$, positive semi-definite and trace one $\mathrm{Tr}[\rho] = 1$. For a given mixed state of an open system, there is a pure state $\rho^{v\oplus a} = |\Psi\rangle\langle\Psi|$ with $|\Psi\rangle = \sum_v \sum_a \Psi(v,a) \left|v\right\rangle \left|a\right\rangle$ through taking into account the environment with the basis $|a\rangle$. In terms of the purification method, every mixed state can be purified in an enlarged Hilbert space such that $\rho = \mathrm{Tr}_a[|\Psi\rangle\langle\Psi|]$. Analogous to previous pure-state ansatz using the RBM, one needs to map a given neural network, named the neural density operator (NDO), with parameters θ to the density operator in Eq.(S6) as (37):

$$\rho_\theta = \sum_{v,v'} \rho_\theta(v,v') \left|v\right\rangle \left\langle v'\right| \tag{S7}$$

To this end, the purification method of the NDO is also used. The environment is now represented by an extra ancillary layer a in addition to the hidden and visible layers in traditional RBM representation, and the basis of the environment Hilbert space is encoded in the number of ancillary neurons. The Ising-type energy of the three-layer neural network is defined as:

$$E_\theta(v,h,a) = \sum_{ij} W_{ij}^\theta h_i v_j + \sum_{jk} U_{jk}^\theta v_j a_k + \sum_j b_j^\theta v_j + \sum_i c_i^\theta h_i + \sum_k d_k^\theta a_k, \tag{S8}$$

where U_θ is the connecting weight matrix between the visible and ancillary layers, and d_θ is the bias vector coupled to the ancillary layer. The purified wavefunction of the NDO takes the form $|\Psi_\theta\rangle = \sum_v \sum_a \Psi_\theta(v,a) \left|v\right\rangle \left|a\right\rangle$. By tracing out the third ancillary layer representing the environment, the NDO is obtained and determined by the neural parameters as:

$$\rho_\theta = \sum_{v,v'} \left[\sum_a \Psi_\theta(v,a)\Psi_\theta^*(v,a)\right] \left|v\right\rangle \left\langle v'\right|. \tag{S9}$$

To represent the amplitude and phase of the purified neural-network state $|\Psi_\theta\rangle$, two different sets of parameters $\lambda = \{W_\lambda, U_\lambda, b_\lambda, c_\lambda, d_\lambda\}$ and $\mu = \{W_\mu, U_\mu, b_\mu, c_\mu, d_\mu\}$ are also needed. In this way, the complex amplitudes of the purified neural-network state can be written as:

$$\Psi_{\lambda\mu}(v,a) = Z_\lambda^{-\frac{1}{2}} \sqrt{p_\lambda(v,a)} e^{i \log p_\mu(v,a)/2} = Z_\lambda^{-\frac{1}{2}} \sqrt{\sum_h e^{-E_\lambda(v,h,a)}} e^{i \frac{\log[\sum_h e^{-E_\mu(v,h,a)}]}{2}}, \tag{S10}$$

where $Z_\lambda = \sum_v \sum_h \sum_a e^{-E_\lambda(v,h,a)}$ is a normalization constant. Then, the matrix elements of the NDO can be obtained

$$\rho_{\lambda\mu}(v,v') = Z_\lambda^{-1} e^{\Gamma_\lambda^+(v,v') + i\Gamma_\mu^-(v,v') + \Pi_{\lambda\mu}(v,v')}, \tag{S11}$$

where,

$$\begin{aligned} \Gamma_\theta^\pm(v,v') &= \frac{1}{2}\Big[\sum_i \log(1 + e^{W_\theta^{[i]} v + c_\theta^{[i]}}) \pm \sum_i \log(1 + e^{W_\theta^{[i]} v' + c_\theta^{[i]}}) + b_\theta^T (v \pm v')\Big], \\ \Pi_{\lambda\mu}(v,v') &= \sum_i \log\{1 + \exp[\frac{1}{2} U_\lambda^{[i]}(v + v') + \frac{i}{2} U_\mu^{[i]}(v - v') + d_\lambda^{[i]}]\}. \end{aligned} \tag{S12}$$

$A^{[i]}$ denotes the i-th row of A, and A^{T} is the transpose of A. The learning of the NDO also involves finding the optimal set of neural parameters $\theta = \{\lambda, \mu\}$ such that the model distribution can perfectly match the measured distribution in a collection of bases $\{v^n\}$, i.e., $P_{\lambda\mu}(v^n) = \rho_{\lambda\mu}(v^n, v^n) \simeq \rho(v^n, v^n) = P(v^n)$. By plugging the optimal neural parameters into Eq.(S11), the target mixed state can be reconstructed.

Section B. Neural-network reconstruction for the open quantum walk.

Model of an open quantum walk. – The dynamics of the open quantum walk (QW) are usually non-unitary, and the density operator is introduced to describe the mixed state, which reads as:

$$\rho = \sum_{s,l;s',l'} \rho_{sl,s'l'} \left|s\right\rangle \left|l\right\rangle \left\langle s'\right| \left\langle l'\right| \quad \text{with} \quad s =\uparrow,\downarrow \quad \text{and} \quad l = 0,1,2,\cdots \tag{S13}$$

The size of the lattice space $|l\rangle$ ($l = 0,1,2,\cdots,N$) is $N+1$ for an N-step open QW. The non-unitary evolution of the open QW can be described by (45):

$$\rho(t+1) = (1 - w_s - w_l)\hat{U}\rho(t)\hat{U}^\dagger + w_s \sum_s \hat{\mathbb{P}}_s \hat{U}\rho(t)\hat{U}^\dagger \hat{\mathbb{P}}_s + w_l \sum_l \hat{\mathbb{P}}_l \hat{U}\rho(t)\hat{U}^\dagger \hat{\mathbb{P}}_l, \tag{S14}$$

where $\hat{U} = \hat{S}\hat{R}$ is a unitary operator. $\hat{\mathbb{P}}_s = \sum_l |s,l\rangle\langle s,l|$ and $\hat{\mathbb{P}}_l = \sum_s |s,l\rangle\langle s,l|$ are the projectors that contribute to the decoherence of the coin and the lattice space, respectively. w_s and w_l are the probabilities of a decoherence event occurring on the coin and lattice, respectively, for each time step, representing the coupling strength between the open QW system and some Markovian environment.

Training datasets. – For an N-step open QW, we can obtain $N_b = 2(N+1)+1$ measurement bases $\{|v^n\rangle\}$ ($n = 0,1,\cdots,2(N+1)$) in our photonic QW setup, where each base $|v^n\rangle$ is composed of $2(N+1)$ linearly independent base vectors. The first base is the reference base $|v^{n=0}\rangle = |s\rangle\,|l\rangle$:

$$\{\left|\uparrow\right\rangle \otimes \left|l\right\rangle, \left|\downarrow\right\rangle \otimes \left|l\right\rangle\}_{l=0}^{N}, \tag{S15}$$

where,

$$\begin{aligned} &|\uparrow\rangle = [1,0]^{\mathrm{T}}, |\downarrow\rangle = [0,1]^{\mathrm{T}},\\ &|l=0\rangle = [1,0,\cdots,0,0]^{\mathrm{T}},\\ &|l=1\rangle = [0,1,\cdots,0,0]^{\mathrm{T}},\\ &\cdots\\ &|l=N-1\rangle = [0,0,\cdots,1,0]^{\mathrm{T}},\\ \text{and } &|l=N\rangle = [0,0,\cdots,0,1]^{\mathrm{T}}. \end{aligned} \tag{S16}$$

The other base can be obtained through the base transformation acting on $|v^0\rangle$. $\left|v^{2k-1}\right\rangle$ and $\left|v^{2k}\right\rangle$ ($k \in \{1,2,\cdots,N+1\}$) are written as $\{\frac{1}{\sqrt{2}}(\left|\uparrow\right\rangle \left|l\right\rangle \pm i \left|\downarrow\right\rangle \left|[l-(k-1)]\mathrm{mod}(N+1)\right\rangle)\}_{l=0}^{N}$ and $\{\frac{1}{\sqrt{2}}(\left|\uparrow\right\rangle \left|l\right\rangle \pm \left|\downarrow\right\rangle \left|[l-(k-1)]\mathrm{mod}(N+1)\right\rangle)\}_{l=0}^{N}$, respectively. In terms of measurement bases

$\{|v^n\rangle\}$, we can define N_b ensembles of projector $\{\hat{\mathbb{P}}_j^n = |v_j^n\rangle\langle v_j^n|\}_j$ ($j = 1, 2, \cdots, 2(N+1)$). For each ensemble of projectors, the normalization condition $\sum_j \hat{\mathbb{P}}_j^n = \mathrm{I}$ (I is the identity matrix of dimensions $4(N+1)^2$) is always satisfied. For each measurement base $|v^n\rangle$, the probability distribution imposed on the target QW state ρ can be obtained by the linear expression $P(v^n) = \{\mathrm{Tr}[\hat{\mathbb{P}}_j^n \rho]\}_j$, with $\sum_{v^n} P(v^n) = 1$. Thus, the training datasets consist of $N_b = 2(N+1)+1$ sets of probability distributions $\{P(v^n)\}$, where each set of distribution $P(v^n)$ on the base $|v^n\rangle$ has $2(N+1)$ real-valued elements satisfying the normalization condition.

Taking a one-step QW as an example, we have five measurement bases $\{|v^n\rangle\}$ ($n = 0, 1, 2, 3, 4$) with each base $|v^n\rangle$ composed of four linearly independent base vectors, which can be written as:

$$|v^0\rangle : \{ \begin{pmatrix}1\\0\\0\\0\end{pmatrix}, \begin{pmatrix}0\\1\\0\\0\end{pmatrix}, \begin{pmatrix}0\\0\\1\\0\end{pmatrix}, \begin{pmatrix}0\\0\\0\\1\end{pmatrix} \}$$

$$|v^1\rangle : \{ \begin{pmatrix}\frac{1}{\sqrt{2}}\\0\\\frac{i}{\sqrt{2}}\\0\end{pmatrix}, \begin{pmatrix}0\\\frac{1}{\sqrt{2}}\\0\\\frac{i}{\sqrt{2}}\end{pmatrix}, \begin{pmatrix}\frac{1}{\sqrt{2}}\\0\\\frac{-i}{\sqrt{2}}\\0\end{pmatrix}, \begin{pmatrix}0\\\frac{1}{\sqrt{2}}\\0\\\frac{-i}{\sqrt{2}}\end{pmatrix} \}, \quad |v^2\rangle : \{ \begin{pmatrix}\frac{1}{\sqrt{2}}\\0\\\frac{1}{\sqrt{2}}\\0\end{pmatrix}, \begin{pmatrix}0\\\frac{1}{\sqrt{2}}\\0\\\frac{1}{\sqrt{2}}\end{pmatrix}, \begin{pmatrix}\frac{1}{\sqrt{2}}\\0\\\frac{-1}{\sqrt{2}}\\0\end{pmatrix}, \begin{pmatrix}0\\\frac{1}{\sqrt{2}}\\0\\\frac{-1}{\sqrt{2}}\end{pmatrix} \} \quad \text{(S17)}$$

$$|v^3\rangle : \{ \begin{pmatrix}\frac{1}{\sqrt{2}}\\0\\0\frac{i}{\sqrt{2}}\end{pmatrix}, \begin{pmatrix}0\\\frac{1}{\sqrt{2}}\\\frac{i}{\sqrt{2}}\\0\end{pmatrix}, \begin{pmatrix}\frac{1}{\sqrt{2}}\\0\\0\\\frac{-i}{\sqrt{2}}\end{pmatrix}, \begin{pmatrix}0\\\frac{1}{\sqrt{2}}\\\frac{-i}{\sqrt{2}}\\0\end{pmatrix} \}, \quad |v^4\rangle : \{ \begin{pmatrix}\frac{1}{\sqrt{2}}\\0\\0\\\frac{1}{\sqrt{2}}\end{pmatrix}, \begin{pmatrix}0\\\frac{1}{\sqrt{2}}\\\frac{1}{\sqrt{2}}\\0\end{pmatrix}, \begin{pmatrix}\frac{1}{\sqrt{2}}\\0\\0\\\frac{-1}{\sqrt{2}}\end{pmatrix}, \begin{pmatrix}0\\\frac{1}{\sqrt{2}}\\\frac{-1}{\sqrt{2}}\\0\end{pmatrix} \}$$

, respectively. Then, the five ensembles of projector $\{\hat{\mathbb{P}}^n\}$ ($n = 0, 1, 2, 3, 4$) are defined as:

$$\{\hat{\mathbb{P}}^0\} = \{ \begin{pmatrix}1&0&0&0\\0&0&0&0\\0&0&0&0\\0&0&0&0\end{pmatrix}, \begin{pmatrix}0&0&0&0\\0&1&0&0\\0&0&0&0\\0&0&0&0\end{pmatrix}, \begin{pmatrix}0&0&0&0\\0&0&0&0\\0&0&1&0\\0&0&0&0\end{pmatrix}, \begin{pmatrix}0&0&0&0\\0&0&0&0\\0&0&0&0\\0&0&0&1\end{pmatrix} \},$$

$$\{\hat{\mathbb{P}}^1\} = \{ \begin{pmatrix}\frac{1}{2}&0&\frac{-i}{2}&0\\0&0&0&0\\\frac{i}{2}&0&\frac{1}{2}&0\\0&0&0&0\end{pmatrix}, \begin{pmatrix}0&0&0&0\\0&\frac{1}{2}&0&\frac{-i}{2}\\0&0&0&0\\0&\frac{i}{2}&0&\frac{1}{2}\end{pmatrix}, \begin{pmatrix}\frac{1}{2}&0&\frac{i}{2}&0\\0&0&0&0\\\frac{-i}{2}&0&\frac{1}{2}&0\\0&0&0&0\end{pmatrix}, \begin{pmatrix}0&0&0&0\\0&\frac{1}{2}&0&\frac{i}{2}\\0&0&0&0\\0&\frac{-i}{2}&0&\frac{1}{2}\end{pmatrix} \},$$

$$\{\hat{\mathbb{P}}^2\} = \{ \begin{pmatrix}\frac{1}{2}&0&\frac{1}{2}&0\\0&0&0&0\\\frac{1}{2}&0&\frac{1}{2}&0\\0&0&0&0\end{pmatrix}, \begin{pmatrix}0&0&0&0\\0&\frac{1}{2}&0&\frac{1}{2}\\0&0&0&0\\0&\frac{1}{2}&0&\frac{1}{2}\end{pmatrix}, \begin{pmatrix}\frac{1}{2}&0&\frac{-1}{2}&0\\0&0&0&0\\\frac{-1}{2}&0&\frac{1}{2}&0\\0&0&0&0\end{pmatrix}, \begin{pmatrix}0&0&0&0\\0&\frac{1}{2}&0&\frac{-1}{2}\\0&0&0&0\\0&\frac{-1}{2}&0&\frac{1}{2}\end{pmatrix} \}, \quad \text{(S18)}$$

$$\{\hat{\mathbb{P}}^3\} = \{ \begin{pmatrix}\frac{1}{2}&0&0&\frac{-i}{2}\\0&0&0&0\\0&0&0&0\\\frac{i}{2}&0&0&\frac{1}{2}\end{pmatrix}, \begin{pmatrix}0&0&0&0\\0&\frac{1}{2}&\frac{-i}{2}&0\\0&\frac{i}{2}&\frac{1}{2}&0\\0&0&0&0\end{pmatrix}, \begin{pmatrix}\frac{1}{2}&0&0&\frac{i}{2}\\0&0&0&0\\0&0&0&0\\\frac{-i}{2}&0&0&\frac{1}{2}\end{pmatrix}, \begin{pmatrix}0&0&0&0\\0&\frac{1}{2}&\frac{i}{2}&0\\0&\frac{-i}{2}&\frac{1}{2}&0\\0&0&0&0\end{pmatrix} \},$$

$$\{\hat{\mathbb{P}}^4\} = \{ \begin{pmatrix} \frac{1}{2} & 0 & 0 & \frac{1}{2} \\ 0 & 0 & 0 & 0 \\ 0 & 0 & 0 & 0 \\ \frac{1}{2} & 0 & 0 & \frac{1}{2} \end{pmatrix}, \begin{pmatrix} 0 & 0 & 0 & 0 \\ 0 & \frac{1}{2} & \frac{1}{2} & 0 \\ 0 & \frac{1}{2} & \frac{1}{2} & 0 \\ 0 & 0 & 0 & 0 \end{pmatrix}, \begin{pmatrix} \frac{1}{2} & 0 & 0 & \frac{-1}{2} \\ 0 & 0 & 0 & 0 \\ 0 & 0 & 0 & 0 \\ \frac{-1}{2} & 0 & 0 & \frac{1}{2} \end{pmatrix}, \begin{pmatrix} 0 & 0 & 0 & 0 \\ 0 & \frac{1}{2} & \frac{-1}{2} & 0 \\ 0 & \frac{-1}{2} & \frac{1}{2} & 0 \\ 0 & 0 & 0 & 0 \end{pmatrix} \}.$$

The codes used to numerically generate the synthetic measurement datasets for various QW models with arbitrary time steps can be found in Ref. (51), using the MATHEMATICA program.

Learning mixed state of open QW. – The goal of neural network tomography of the QW is to find the optimal approximation for the target density matrix, using the measurement datasets obtained by numerically or experimentally. Specially, in total, we have $N_b = 2(N+1)+1$ sets of probability distributions $\{P(v^n)\}$ $(n = 0, 1, \cdots, 2(N+1))$ as the training datasets of the neural network. The model distribution $P_{\lambda\mu}(v^n) = \rho_{\lambda\mu}(v^n, v^n)$ is fitted to the measurement distribution by a proper cost function. The cost function we use is defined as the total statistical distance:

$$\mathcal{D}_{\lambda\mu} = \sum_n \sum_{v^n} P(v^n) \log[P(v^n)/P_{\lambda\mu}(v^n)], \tag{S19}$$

which can be interpreted as a measurement of the distance between the model and the measurement distribution. Thus, when $\mathcal{D}_{\lambda\mu} \to 0$, the model distribution predicted by the neural-network ansatz approximately equals to the measurement distribution on the target state. We minimize the value of the cost function over all the components of the given measurement datasets to update the network weights and biases $\{W_{\lambda\mu}, U_{\lambda\mu}, b_{\lambda\mu}, c_{\lambda\mu}, d_{\lambda\mu}\}$, using a gradient-based optimization. Herein, we utilize our self-developed generalized natural gradient descent (GNGD) that is of core importance in the mixed-state learning work, since it has been numerically shown that it has better performance than some conventional optimizers (38). At each iteration i, the GNGD procedure updates the neural parameters in the form:

$$\theta_{i+1} = \theta_i - \eta_i G^{-1} \nabla \mathcal{D}_\theta. \tag{S20}$$

Here, η_i represents the learning rate that is determined by the line search process. G is the metric on the cost function and should be chosen carefully to ensure the better performance of the GNGD optimizer. We choose an identity matrix as the metric of the flat reference space $G_{ij}^{\mathrm{ref}} = \delta_{i,j}$ and obtain the well-performed metric G through the conversion of coordinates.

All the training above for the open QW is implemented in a FORTRAN program, and the corresponding code can be found in Ref. (51). In each iteration process for the current experimental work, we directly sum over all the terms in $\mathcal{D}_\theta$ for being free of the sampling error of Monte Carlo sampling. The criterion used to stop the training is that the norm of the gradient reaches a fixed value of 10^{-8}. For the model hyperparameters, the number of visible neurons is uniquely determined by the dimension of the basis $|v^{n=0}\rangle = |s\rangle\,|l\rangle$. The number of hidden and ancillary neurons varies as the correlation within the physical systems and the one between the system and the environment become more complex, which is qualitatively determined by the number of time steps and the coupling strength between the system and the environment.

Section C. Benchmarking neural-network reconstruction of an open QW with depolarizing noise.
In this section, we consider the open QW under depolarizing noise, where the target density matrix for each time step is:

$$\rho_t = (1 - p_{\rm de})(\prod_t \hat{U}_t)\rho(0)(\prod_t \hat{U}_t)^\dagger + \frac{p_{\rm de}}{d}\mathrm{I}. \tag{S21}$$

$p_{\rm de} \in [0,1]$ denotes the depolarizing noise strength, $d = 2(N+1)$ and I is the identity matrix of dimension $4(N+1)^2$ for an N-step open QW. To benchmark the NDO tomography of this type of open QW, we set the initial state to $\rho_0 = |\psi_0\rangle\langle\psi_0|$ with $|\psi_0\rangle = \frac{1}{\sqrt{2}}(|\uparrow\rangle + i\,|\downarrow\rangle) \otimes |0\rangle$. The choice of the coin-flip angle $\alpha \in [0,\pi)$ is completely random for each time step, and then the unitary operator $\hat{U}_t = \hat{S}\hat{R}(\alpha_t)$ is time dependent. We generate 100 samples of the open QW with different degrees of decoherence determined by the noise strength $p_{\rm de} \in [0,1]$ for each time step. Fig. S1(A) show the reconstruction results obtained using NDO and GNGD optimizer for the open QW with 3000 samples in total. The reconstruction fidelity can still be a value of $\sim 93.5\%$ with the partial measurements $\{P(v^n)\}$ defined in the previous section B. The mean value of the reconstruction errors for the purity is lower than 6×10^{-3}, which indicates a close agreement with the target density matrix. Here, the number of the hidden and ancillary neurons of the NOD is set to 10 during the training process. The number of the visible neurons is uniquely determined by the dimension of the reference base $|v^{n=0}\rangle = |s\rangle\,|l\rangle$ for each time step of QW. We emphasize that the mean value of the reconstruction fidelity for the QW with depolarizing noise is slightly lower than the result of open QW with phase decoherence of the coin ($\sim 97.5\%$) in the main text. This is because the mixed-state matrix of QW with depolarizing noise could be a full-rank matrix ($p_{\rm de} = 1$), which demands more measurement settings.

For comparison, we also perform the maximum likelihood estimation (MaxLik) on the 3000 samples. The MaxLik estimator we use takes the form:

$$\rho_{\rm m} = TT^\dagger / \mathrm{Tr}(TT^\dagger), \tag{S22}$$

which can give a “physical” density matrix that is Hermitian, positive semi-definite and trace one, inspired by the Cholesky decomposition. The matrix T is a lower triangular matrix with diagonal elements being real-valued and non-diagonal elements being complex-valued. For an N-step open QW, $4(N+1)^2$ real numbers need to be optimized such that the MaxLik can give a distribution $P_{\rm m}(v^n) = \{\mathrm{Tr}[\hat{\mathbb{P}}^n_j \rho_{\rm m}]\}_j$ approximately equal to the measurement distribution$P(v^n) = \{\mathrm{Tr}[\hat{\mathbb{P}}^n_j \rho]\}_j$ ($n = 0, 1, \cdots, 2(N+1)$) in terms of the total statistical distance as the cost function. The values of the $4(N+1)^2$ real numbers are randomly initialized and updated using the conjugate gradient (CG) algorithm (55). The stopping criterion is that the norm of the gradient reaches a fixed value of 10^{-8}. The MaxLik reconstruction fidelity and purity error are shown in Fig. S1(B). For the open QW in a depolarizing channel, it is also obvious that the power of the NDO learning method (mean value of fidelity >0.935 and purity error <0.006) is superior to that of the standard MaxLik method (mean value of fidelity >0.89 and purity error <0.03) when the given measurement is limited and partial.

Section D. Relation between the fluctuating degree and the mixing parameter.

In this section, we provide an empirical relation between the fluctuating degree $\delta\beta \in [0, \pi]$ and the mixing parameter $w = w_s + w_l \in [0, 1]$ in Eq. (S14). For introducing decoherence into the isolated QW, one common method shown in Eq.(S14) is to add extra measurement operators $\hat{\mathbb{P}}_s$ ($s =\uparrow, \downarrow$) and $\hat{\mathbb{P}}_l$ ($l = 0, 1, 2, \cdots, N$) to the coin and position space, respectively. However, this measurement-based decoherence will bring in the inevitable loss in photonic QW experiments. In the main text, we adopt an alternative method instead of projection to realize the open QW in which an additional phase gate $\hat{R}(\beta) = e^{\frac{i}{2}\beta\hat{\sigma}_z}$ with a fast fluctuating phase $\beta \in [-\delta\beta, \delta\beta]$ is added at each step. Then, the dynamics of the open QW with phase decoherence of the coin can be written as:

$$\rho_t = \sum_j \hat{U}\hat{R}(\beta_j)\rho(0)\hat{R}(\beta_j)^\dagger \hat{U}^\dagger, \tag{S23}$$

where $\hat{U} \equiv \hat{S}\hat{R}(\pi/4)$ corresponds to the Hadamard walk as an example. For each trial j, the phase β_j is chosen randomly from the set $[-\delta\beta, \delta\beta]$. By averaging over sufficient trials, the QW can be transformed to the classical random walk when $\delta\beta = \pi$. Thus, the two approaches are equivalent in the classical limit, as shown in Fig. S2, taking a five-step open Hadamard QW as an example. The result (i.e., the purity of the open QW) obtained by the phase is compared with the one predicted by the open QW model in Eq. (S14). Fig. S2 shows the empirical relation between the fluctuating degree $\delta\beta \in [0, \pi]$ and the mixing parameter $w = w_s + w_l \in [0, 1]$ that the system's purity obtained for a certain value of the mixing parameter w can be faithfully reproduced with a proper value of the fluctuating degree $\delta\beta$ in the experimental photonic QW model.

Fig. S1.

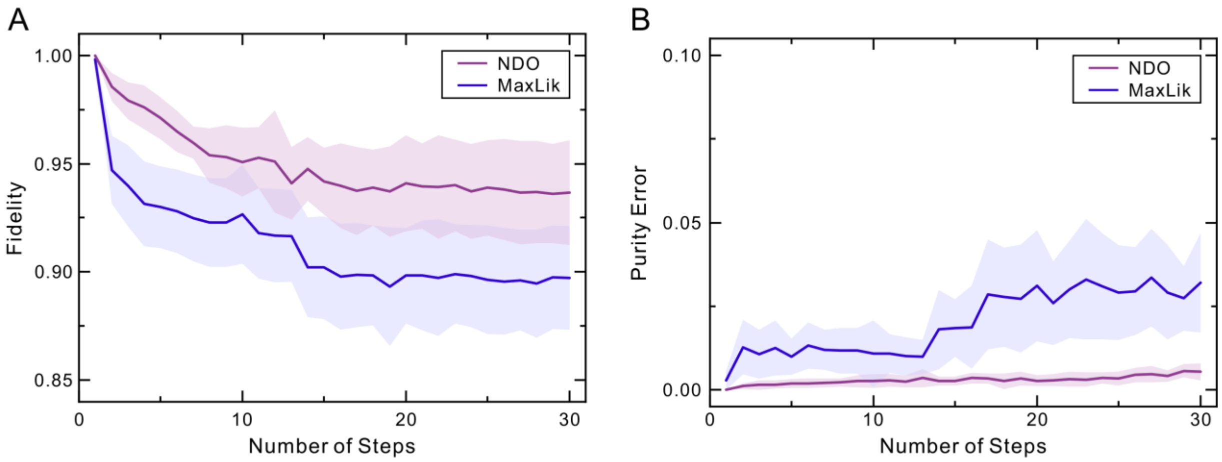

Fig. S1 Benchmarking NDO and MaxLik tomography of the open QW with depolarizing noise. (A) NDO (red line) and MaxLik (blue line) reconstruction fidelity as a function of the number of time steps for QW with depolarizing noise. The colored shaded regions are the standard errors with 100 random samples for each time step, and the solid lines are the averaging results. **(B)** shows the error in the purity of the reconstructed mixed state using the NDO and MaxLik methods.

Fig. S2.

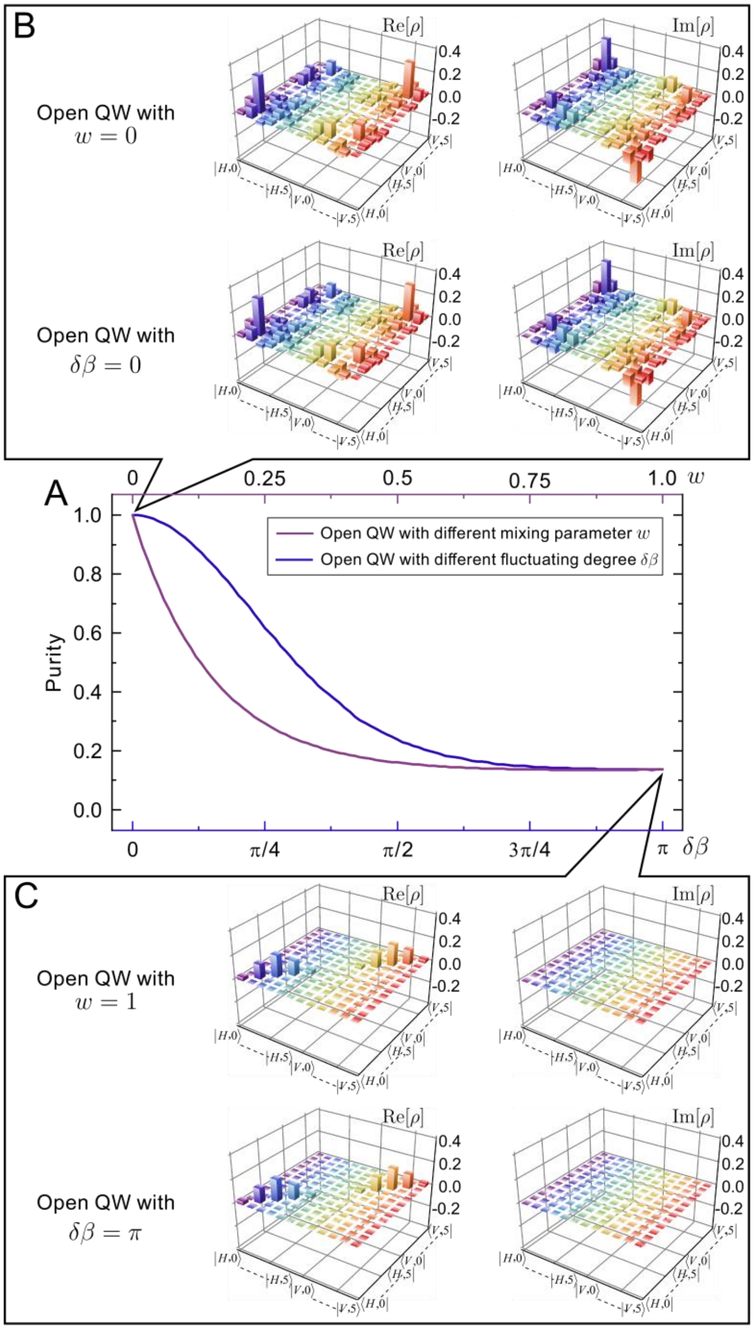

Fig. S2. Comparison of the fluctuating degree and the mixing parameter. (A) Purity of the open QW as a function of the mixing parameter $w = w_s + w_l$ (red solid line) and the fluctuating degree $\delta\beta$ (blue solid line). **(B)** Real and imaginary parts of the density matrix for the open QW with $w = 0$ (upper panels) and $\delta\beta = 0$ (lower panels). (C) Real and imaginary parts of the density matrix for the open QW with $w = 1$ (upper panels) and $\delta\beta = \pi$ (lower panels). In the numerical simulations, we set $w_s = w_l$ and the number of trials is 1500.
